# Genetic diversity and antibiotic resistance of *Shigella* spp. isolates from food products

**DOI:** 10.1002/fsn3.2603

**Published:** 2021-09-23

**Authors:** Babak Pakbin, Zahra Amani, Samaneh Allahyari, Shaghayegh Mousavi, Razzagh Mahmoudi, Wolfram Manuel Brück, Amir Peymani

**Affiliations:** ^1^ Medical Microbiology Research Center Qazvin University of Medical Sciences Qazvin Iran; ^2^ Institute for Life Technologies University of Applied Sciences Western Switzerland Valais‐Wallis Switzerland

**Keywords:** antimicrobial resistance, food samples, genetic diversity, *Shigella* species

## Abstract

The emergence of multidrug‐resistant *Shigella* is a significant threat to global public health. Limited studies have investigated the incidence, antimicrobial susceptibility, and genetic diversity of *Shigella* isolated from food products. Conventional culture‐based, serologic, molecular, disk diffusion, PCR, and RAPD‐PCR methods were used to determine the prevalence rate, phenotypic and genotypic antibiotic resistance profile, and genetic diversity of the *Shigella* isolates from food samples including vegetable salad, ground meat, and raw cow's milk (405 samples). The prevalence rate of *Shigella* in food samples was 4.44%. The incidence of *S. sonnei* (3.7%) was higher than that of *S. flexneri* (0.74%). *S. dysenteriae* and *S. boydii* were not detected in food samples examined. Also, no *Shigella* were recovered from raw cow's milk. This study showed that the *Shigella* isolates were resistant to sulfamethoxazole/trimethoprim (83.3%), amoxicillin (66.6%), streptomycin (66.6%), tetracycline (61.1%), ampicillin (50%), amoxicillin–clavulanic acid (50%), azithromycin (50%), and chloramphenicol (50%) and completely sensitive to cefoxitin, cefepime, amikacin, and gentamicin. All *Shigella* isolates were multidrug‐resistant. We detected *bla*_SHV_ resistance gene in all isolates; however, no isolate harbored *bla*_TEM_ gene. RAPD‐PCR categorized the *Shigella* isolates into five main clusters. The highest antibiotic resistance was observed in the isolates of cluster R4. The finding of this study also indicated an association between antimicrobial resistance profiles and genotyping properties of the isolates. Novel food monitoring systems, including surveillance of multidrug‐resistant foodborne pathogens, especially in developing countries, are required to control the foodborne diseases.

## Graphical abstract

*Shigella* spp. can be isolated from food samples, cause foodborne illness, and are responsible for serious outbreaks worldwide. Limited studies have investigated the incidence, antimicrobial susceptibility, and genetic diversity of *Shigella* spp. isolated from food products. In this study, we determined the prevalence rate, antimicrobial resistance patterns, and genetic diversity of the *Shigella* isolates from food samples including vegetable salad, ground meat, and raw cow's milk. We observed that all isolates were multi‐drug resistant. The findings of this study also indicated an association between antimicrobial resistance profiles and genotyping of the *Shigella* isolates. This is the first study that showed an association between antimicrobial resistance phenotypes and genotyping of *Shigella* spp. isolated from food.

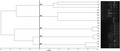

## 1 INTRODUCTION

Foodborne pathogens (including bacteria, viruses, and parasites) have become one of the most critical public health concerns around the world. When a pathogen is ingested with the contaminated food or drink and establishes itself in the human host or the toxin of a toxigenic pathogen is released and ingested, a foodborne illness occurs (Nyachuba, 2010). WHO estimated more than 600 million cases and 420 000 deaths each year caused by 22 major foodborne diseases worldwide (Organization, 2021). The most severe foodborne illnesses tend to occur in immunocompromised, very young, or very old patients. Foodborne pathogens (mainly bacterial agents), leading to intestinal and extraintestinal diseases, are causing significant adverse effects on human health and economic hardship (Hanson et al., 2012). The most common foodborne pathogenic bacteria include *Escherichia coli*, *Campylobacter* spp., *Salmonella* spp., *Clostridium perfringens*, *Staphylococcus aureus*, *Shigella* spp., and *Listeria monocytogenes*. More than 200 intestinal and extraintestinal disorders have been identified to be caused by foodborne pathogens (Pakbin et al., 2020; Smith & Fratamico, 2018).

Shigellosis, caused by *Shigella* spp., is characterized by invasion of the epithelial cells lining the colon and rectum. *Shigella* is a non‐motile, rod‐shaped, non‐spore‐forming, and gram‐negative bacteria belonging to the *Enterobacteriaceae* family. This pathogen includes four species: *S*. *dysenteriae* (serogroup A), *S. flexneri* (serogroup B), *S. boydii* (serogroup C), and *S. sonnei* (serogroup D). Detection of *S. sonnei* and *S. flexneri* is more frequent in developed and developing countries, respectively. It is also one of the most critical causes of diarrhea‐related mortality and morbidity around the world. It is estimated that 55,000 and 110,000 deaths and hospitalizations, respectively, in children under five years old and older children or adults worldwide, are caused by *Shigella* annually (Bennish & Ahmed, 2020). *Shigella* spp., as the causative agent of bacillary dysentery, has been involved in several foodborne and waterborne outbreaks (Kotloff et al., 2018).

*Shigella* is the third most reported bacterial foodborne pathogen. Foods are mainly contaminated with these pathogens by infected food handlers with poor personal hygiene (Nataro et al., 2011). However, foodborne outbreaks caused by *Shigella* mainly occur while foods are subjected to preparation or processing by hands, exposed to insufficient thermal processing, and delivered or served raw to the consumers. *Shigella* can survive in acidic and salty conditions, be transmitted to the human host and cause disease (Baker & The, 2018). Fresh vegetables, deli meats, and unpasteurized milk are more susceptible to be contaminated with *Shigella* spp. (Warren et al., 2006). Some researches also demonstrated that *Shigella* spp. had been isolated from ground beef, raw oysters, bean dip, raw vegetables, potato salad, and fish (Ahmed & Shimamoto, 2014, 2015; Bantawa et al., 2019; Cetinkaya et al., 2008).

In addition to the prevalence rate, the multidrug resistance of *Shigella* spp. has recently been considered a significant concern in food safety. Antibiotic therapy reducing the likelihood of complications, death, and hastening clinical recovery is the primary treatment of shigellosis (Ingle et al., 2020). The emergence of multidrug‐resistant (MDR) *Shigella* spp. makes the treatment of shigellosis more difficult and highlights the problem of antimicrobial resistance (Ma et al., 2018). However, it is essential to determine the appropriate antibiotics for the treatment of shigellosis via a deep understanding of the current changes in resistance patterns by studying the phenotypic and genotypic antimicrobial resistance profiles of the *Shigella* isolates. Molecular characterization of MDR strains has been used to analyze isolated pathogens. *Shigella* isolates have also been used to determine the genetic relatedness and diversity between the isolates (Zamanlou et al., 2018). Random amplified polymorphic DNA (RAPD) is one of the PCR‐based molecular techniques for genotyping and determining genetic diversity between the pathogenic isolates (Ben Braïek et al., 2018). Several studies have been implemented to characterize the antibiotic resistance in *Shigella* isolates from clinical specimens; however, limited literature provides the molecular characterization of MDR *Shigella* isolates from food samples (Shahin et al., 2019). Therefore, the purpose of this study was to investigate the genetic relatedness and molecular characterization of MDR *Shigella* isolated from fresh vegetable, unpasteurized milk, and ground meat samples collected in a survey in Iran.

## 2 MATERIALS AND METHODS

### 2.1 Food samples collection

Food samples (*N* = 405), including 135 raw cow's milk, 135 vegetable salads, and 135 ground meat samples, were randomly collected from different local supermarkets and restaurants in various areas of Qazvin city, Iran, between July and December 2018. All samples were aseptically collected in sterile containers and tubes, labeled and immediately transferred to ice‐boxes, and transported to the laboratory for microbiological analysis.

### 2.2 Isolation, biochemical, and serogroup identification of *Shigella* spp

**FIGURE 1:**
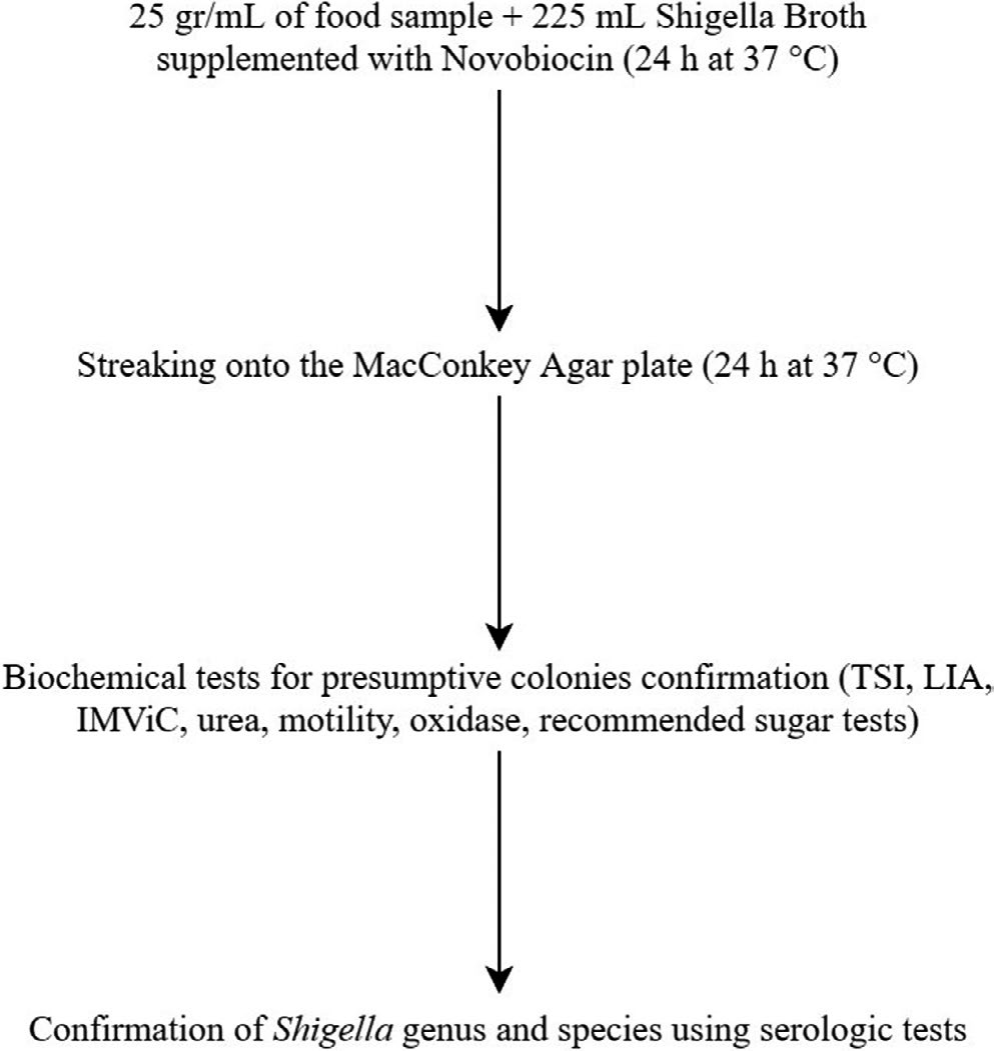
Flowchart of culture‐based methods for detection and identification of presumptive *Shigella* spp. isolated from food samples

*Shigella* spp. were isolated and identified from the food samples according to the method for isolation of *Shigella* from food described previously (Mokhtari et al., 2012) (Figure 1). 25 g of each vegetable salad and ground meat samples and 25 ml of raw cow milk samples were mixed with Shigella Broth (Merck, Germany) containing 0.5 µg/ml novobiocin (Sigma‐Aldrich, NSW, Australia), homogenized for 1 min at 320 rpm in a Stomacher BagMixer (InterScience, France), and incubated anaerobically overnight at 42°C. 0.1 ml of enriched samples was streaked onto the MacConkey agar (Merck, Germany) plate and incubated anaerobically at 42°C for 48 hr. Presumptive colonies (convex colorless to slightly pink) on MacConkey agar were selected and subjected for Gram staining and biochemical evaluation, including indole production, citrate utilization, methyl red, Voges‐Proskauer, triple sugar iron, motility, urea, and oxidase (Merck, Germany) (Shahin et al., 2019). All isolates which biochemical tests had confirmed were subjected to serologic tests. Genus and species of the isolates were confirmed and identified by slide agglutination method using *Shigella* Genus and *Shigella* Species Difco antisera kits (BD‐Difco Co., USA) according to the manufacturers` instructions. All *Shigella* isolates were molecularly identified after DNA extraction by conventional PCR to detect the *ipaH* gene using specific primers, including ipaHF: 5'‐GTTCCTTGACCGCCTTTCCGATACCGTC‐3' and ipaHR: 5'‐GCCGGTCAGCCACCCTCTGAGAGTAC‐3'. The PCR mixtures containing 12.5 µl of PCR master mix (Ampliqon, Denmark), 1 µl of each primer (5 µM/µL), 2 µl of DNA template (50 µg/µl), and deionized DNase free water up to the final reaction volume were subjected to initial denaturation at 95°C for 5 min, 40 cycles of amplification (95°C for 30 s, 60°C for 30 s and 72°C for 45 s), and final extension step at 72°C for 5 min. PCR products were characterized using electrophoresis through a 1.2% (w/v) agarose gel stained with DNA safe stain at 90 V for 1 hr and visualized using NovinPars Gel Doc system (NovinPars Co., Iran). *S. dysenteriae* type 1 (ATCC 13313), *S. flexneri* (ATCC 12022), *S. boydii* (ATCC 9207), and *S. sonnei* (ATCC 9290) strains were used as positive controls. All control strains were activated by inoculation into Bovine Heart Infusion (BHI, Merck, Germany) broth and incubation anaerobically at 37°C for 24 hr.

### 2.3 Phenotypic and genotypic antibiotic resistance profile

Antimicrobial susceptibility phenotypes of *Shigella* isolates were determined by the Kirby–Bauer disk diffusion method according to the interpretive criteria and standards previously described by the Clinical and Laboratory Standards Institute (Weinstein & Lewis, 2020). The following tested antimicrobial disks (Oxoid, UK) were used: cefoxitin (FOX), 30 µg; imipenem (IPM), 10 µg; amoxicillin (AMX), 25 µg; ampicillin (AMP), 10 µg; cefepime (FEP), 30 µg; amoxicillin–clavulanic acid (AMC), 20/10 µg; streptomycin (SPT), 10 µg; kanamycin (KAN), 30 µg; amikacin (AMK), 30 µg; gentamicin (GEN), 10 µg; nalidixic acid (NAL), 30 µg; norfloxacin (NOR), 10 µg; levofloxacin (LVX), 5 µg; azithromycin (AZM), 15 µg; tetracycline (TET), 30 µg; colistin (CST) 10 µg; chloramphenicol (CHL), 30 µg; nitrofurantoin (NIT), 300 µg and trimethoprim–sulfamethoxazole (SXT), 1.25/23.75 µg. These phenotypic resistances were selected to be evaluated because they were frequently observed mainly among the *Shigella* and other *Enterobacteriaceae* family strains isolated from clinical and food samples previously. All results were recorded and interpreted based on CLSI guidelines (Weinstein & Lewis, 2020). To investigate the genotypic profile of antibiotic resistance, beta‐lactamase resistance genes including *bla*_TEM_ *, bla*_OXA_ *, bla*_SHV_ *, bla*_CTX‐M‐1_ *, bla*_CTX‐M‐2_ *, bla*_CTX‐M‐8_, and *bla*_CTX‐M‐9_ were detected among 18 *Shigella* isolates by conventional PCR using the specific primers and thermal cycling programs as previously described by Dallenne et al. (2010). The reference strains *Escherichia coli* (ATCC 25922), *Klebsiella pneumoniae* (ATCC 700603), and *Staphylococcus aureus* (ATCC 25923) were included as quality controls.

### 2.4 DNA extraction

All *Shigella* isolates were grown anaerobically on BHI broth overnight at 42°C. 1 ml of the bacterial suspension was suspended in 1 ml of Phosphate‐Buffered Saline (PBS, Promedia Spain) and centrifuged at 10,000 g for 5 min. After removing the supernatant, the bacterial pellet was subjected to DNA extraction using the Sinaclon Gram‐negative DNA extraction kit (Sinaclon Co., Iran) according to the kit manufacturers` instructions. The quality and quantity of the extracted genome were determined using NanoDrop 8000 spectrophotometer (Wilmington, DE, USA). All extracted DNA concentrations were adjusted to 50 μg/ml with PBS before PCR reactions.

### 2.5 RAPD‐PCR genotyping

The genetic relatedness among the *Shigella* isolates was evaluated using the RAPD‐PCR method as previously described. As previously recommended, the arbitrary primer UBC245 (5'‐ CGCGTGCCAG −3') (Berthold‐Pluta et al., 2017) was used in this study for RAPD‐PCR. The total volume of PCR reaction was 25 µl, including 12.5 µl of PCR Master Mix (Ampliqon, Denmark), 1 µl of RAPD primer (5 pmol/µL), 2 µl of DNA template, and sterile deionized water to reach the final reaction volume. The PCR was performed as follows: 94°C for 7 min, 36°C for 1 min, 72°C for 4 min and 40 cycles of 94°C for 1min, 36°C for 1 min and 72°C for 4 min. The amplified products were characterized using electrophoresis through a 1.5% (w/v) agarose gel stained with DNA safe stain at 100 V for 1.5 hr. Gels were visualized, and the patterns were recorded using the NovinPars Gel Doc system (NovinPars Co., Iran). RAPD genetic markers were analyzed using PyElph software (Pavel & Vasile, 2012). The dendrogram was constructed using the Unweighted Pair Group Method with Arithmetic averages (UPGMA) method and Dice coefficient using NTSYS‐pc software version 2.1 (Rohlf, 2001). The RAPD‐PCR pattern types of the isolates with a high similarity index (≥ 0.6) were considered the closely related RAPD pattern type.

### 2.6 Statistical analysis

Chi‐square and Fisher's exact tests were used to evaluate significant differences (*p* <.05) between the contamination rates using IBM SPSS software, version 21.0.1 (IBM Corp., Armonk, NY, USA). All experimental and measurements were performed in triplicate (Table 1).

**TABLE 1:** Antibiotic resistance phenotypes of *Shigella* spp. isolated from vegetable salad and ground meat samples

Antimicrobial class/agent	*n* (%)		
	Vegetable salad (*n* = 13)	Ground meat (*n* = 5)	Total (*n* = 18)
β‐Lactams			
FOX	0 (0)	0 (0)	0 (0)
IPM	2 (15.3)	1 (20.0)	3 (16.6)
AMX	9 (69.2)	3 (60.0)	12 (66.6)
AMP	6 (46.1)	3 (60.0)	9 (50.0)
FEP	0 (0)	0 (0)	0 (0)
AMC	7 (53.8)	2 (40.0)	9 (50.0)
Aminoglycosides			
SPT	9 (69.2)	3 (60.0)	12 (66.6)
KAN	2 (15.3)	1 (20.0)	3 (16.6)
AMK	0 (0)	0 (0)	0 (0)
GEN	0 (0)	0 (0)	0 (0)
Quinolones and fluoroquinolones			
NAL	2 (15.3)	1 (20.0)	3 (16.6)
NOR	2 (15.3)	2 (40.0)	4 (22.2)
LVX	2 (15.3)	1 (20.0)	3 (16.6)
Macrolides			
AZM	6 (46.1)	3 (60.0)	9 (50.0)
Tetracyclines			
TET	7 (53.8)	4 (80.0)	11 (61.1)
Lipopeptides			
CST	2 (15.3)	1 (20.0)	3 (16.6)
Phenicols			
CHL	6 (46.1)	3 (60.0)	9 (50.0)
Nitroheterocyclics			
NIT	2 (15.3)	1 (20.0)	3 (16.6)
Folate pathway antagonists			
SXT	11 (84.6)	4 (80)	15 (83.3)

Abbreviations: AMC, amoxicillin–clavulanic acid; AMK, amikacin; AMP, ampicillin; AMX, amoxicillin; AZM, azithromycin; CHL, chloramphenicol; CST, colistin; FEP, cefepime; FOX, cefoxitin; GEN, gentamicin; IPM, imipenem; KAN, kanamycin; LVX, levofloxacin; NAL, nalidixic acid; NIT, nitrofurantoin; NOR, norfloxacin; SPT, streptomycin; SXT, trimethoprim–sulfamethoxazole; TET, tetracycline.

## 3 RESULTS

### 3.1 Isolation and identification of *Shigella* spp. in food products

**FIGURE 2:**
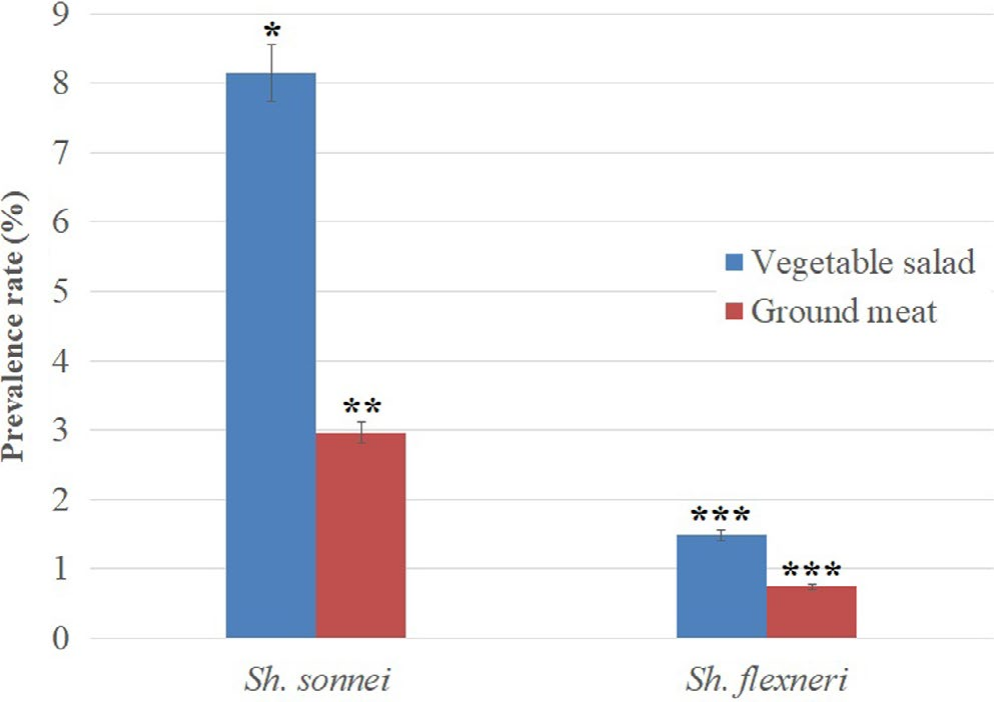
Prevalence rates of *Shigella* spp. in different food sample. *, ** and *** indicates significant differences (*p* =.01)

Prevalence rates of *Shigella* spp. in food products, including vegetable salad and ground meat samples, are shown in Figure 2; however, no *Shigella* spp. were isolated from raw cow's milk samples. In total, eighteen isolates of *Shigella* spp. (4.44%) were detected and molecularly identified in 405 vegetable salad, raw cow milk, and ground meat samples tested in this study. *ipaH* gene was detected in all 18 *Shigella* isolates. Among the isolates, 13 (3.20%) and 5 (1.23%) isolates were detected in vegetable salad and ground meat samples, respectively. Notably, 15 (3.70%) and 3 (0.74%) out of all *Shigella* isolates were identified as *S. sonnei* and *S*. *flexneri,* respectively. No *S. boydii* and *S. dysenteriae* were recovered from the food samples. *Shigella* spp. was detected in 9.62% (13 of 135) of vegetable salad samples, whereas 3.70% (5 of 135) ground meat samples were positive for *Shigella*. Among isolates from vegetable samples, 84.61% (11 of 13) and 15.38% (2 of 13) were identified as *S. sonnei* and *S. flexneri*, respectively. Also, of the ground meat isolates, 80% (4 of 5) were positive for *S. sonnei,* and 20% (1 of 5) were positive for *S. flexneri*. All isolates were primarily identified using biochemical methods and then confirmed by serologic tests. *S. sonnei* strains showed a significantly (*p* =.01) higher prevalence than other species. Also, *Shigella* spp. were detected significantly (*p* =.01) higher in vegetable salad samples than the other food products assessed.

### 3.2 Antibiotic resistance phenotypic and genotypic profile of *Shigella* isolates

All 18 *Shigella* spp. isolates were evaluated for their antimicrobial susceptibility against nine different classes and nineteen different antibiotics. The results of antimicrobial susceptibility testing of the isolates are presented in Table 2. Trimethoprim–sulfamethoxazole (15 isolates; 83.3%), amoxicillin (12 isolates; 66.6%), streptomycin (12 isolates; 66.6%), tetracycline (11 isolates; 61.1%), ampicillin (9 isolates; 50%), amoxicillin–clavulanic acid (9 isolates; 50%), azithromycin (9 isolates; 50%), and chloramphenicol (9 isolates; 50%) resistance were the dominant phenotypes among the *Shigella* isolates. On the other hand, the lowest antimicrobial resistance was observed against norfloxacin (4 isolates; 22.2%), imipenem (3 isolates; 16.6%), kanamycin (3 isolates; 16.6%), nalidixic acid (3 isolates; 16.6%), levofloxacin (3 isolates; 16.6%), colistin (3 isolates; 16.6%), and nitrofurantoin (3 isolates; 16.6%). Also, all isolates were completely susceptible to cefoxitin, cefepime, amikacin, and gentamicin. All the 18 *Shigella* spp. isolates were observed MDR, resistant to 3 or more different classes of antibiotic agents. No significant difference was observed between the antimicrobial susceptibility pattern of the *Shigella* spp. isolates from vegetable salad and ground meat samples. This study detected beta‐lactamase genes in *Shigella* isolates using the conventional PCR method (Table 2). *bla*_SHV_ gene was detected in all 18 *Shigella* isolates; however, no isolates harbored *bla*_TEM_ gene. *bla*_OXA_, *bla*_CTX‐M‐1_, and *bla*_CTX‐M‐8_ resistance genes were detected in 33.3, 33.3, and 33.3% of the isolates, respectively. No significant difference was also seen between the antibiotic resistance gene patterns of *Shigella* isolates from vegetable salad and ground meat samples.

**TABLE 2:** Antibiotic resistance genes in *Shigella* spp. isolated from vegetable salad and ground meat samples

Gene type	*n* (%)		
	Vegetable salad (*n* = 13)	Ground meat (*n* = 5)	Total (*n* = 18)
*bla*_OXA_	2 (40)	4 (30.7)	6 (33.3)
*bla*_SHV_	5 (100)	13 (100)	18 (100)
*bla*_TEM_	0 (0)	0 (0)	0 (0)
*bla*_CTX‐M−1_	2 (40)	4 (30.7)	6 (33.3)
*bla*_CTX‐M−2_	1 (20)	2 (15.3)	3 (16.6)
*bla*_CTX‐M−8_	1 (20)	5 (38.4)	6 (33.3)
*bla*_CTX‐M−9_	0 (0)	3 (23.0)	3 (16.6)

### 3.3 Genetic relatedness among the *Shigella* isolates

**FIGURE 3:**
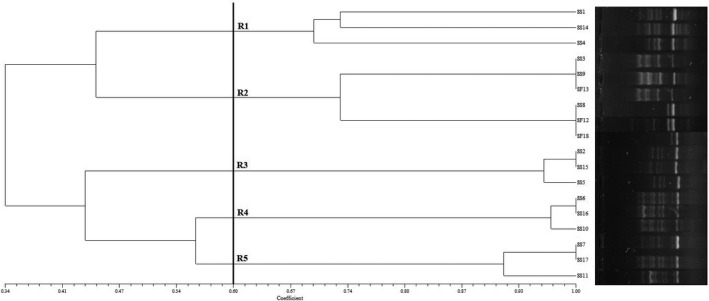
The UPGMA Dendrogram with the Dice coefficient of *Shigella* spp. isolates in the basis of RAPD‐PCR patterns

In this study, genetic relatedness and genotypic polymorphism of eighteen *Shigella* spp. isolates from vegetable salad and ground meat samples were characterized by the RAPD‐PCR method using the UBC245 arbitrary primer. Amplification of the *Shigella* spp. isolates with the UBC245 primer resulted in polymorphic patterns composed of three to eight bands ranging in size from 300 to >1,500 bp (Figure 3). As it is illustrated in Figure 3, the UBC245 oligonucleotide discriminated the eighteen *Shigella* isolates into five clusters (R1–R5) of genetically identical *Shigella* isolates with more than 60% RAPD‐PCR profile similarity (at 60% similarity cut‐off value). The *t* test compared all the groups, and the significant differences were not observed (*p* >.01). Genetic diversity within *Shigella* isolates was calculated using Simpson's diversity index. The average diversity index for the isolates was 0.82. Consequently, a high level of genetic diversity was observed among the *Shigella* spp. isolates. Clusters R1, R3, R4, and R5 contained 3 *Shigella* isolates; however, only cluster R2 included six isolates. All *S. flexneri* isolates were grouped in cluster R2 (Table 3). Vegetable salad *Shigella* spp. isolates were not grouped in the same cluster; ground meat isolates were also included in different clusters. As shown in Table 3, the highest and lowest antibiotic‐resistant isolates were grouped in the clusters R4 and R2, respectively. Antibiotic resistance profiles of the isolates in the same cluster were significantly close to each other. There is also a strong association between the presence of beta‐lactamase genes and the resistance phenotype profile of the isolates. More beta‐lactamase genes were detected in the *Shigella* isolates which were resistant to beta‐lactam antibiotics, including amoxicillin, ampicillin, and amoxicillin–clavulanic acid.

**TABLE 3:** Resistance phenotype, presence of *ipaH* gene, RAPD genotype groups, incidence of beta‐lactamase genes and serogroups in *Shigella* isolates from vegetable salad and ground meat samples

No.	Isolate	Spp.	*ipaH*	Serogroup	Food sample	Resistance phenotype	Resistance genes	RAPD type
1	SS1	*S. sonnei*	+	D	Vegetable salad	AZM, SPT, AMC, AMX, AMP, TET, CHL, SXT	S, C8, C9	R1
2	SS2	*S. sonnei*	+	D	Vegetable salad	AZM, IMP, TET, CHL, SXT	S	R3
3	SS3	*S. sonnei*	+	D	Vegetable salad	SPT, AMC, AX, KAN, SXT	S	R2
4	SS4	*S. sonnei*	+	D	Vegetable salad	AZM, SPT, AMC, AMX, AMP, TET, CHL, SXT	S, C8, C9	R1
5	SS5	*S. sonnei*	+	D	Vegetable salad	AZM, IPM, CHL, SXT	S	R3
6	SS6	*S. sonnei*	+	D	Vegetable salad	NAL, AMC, KAN, AMX, NOR, AMP, TET, LVX, CHL, SXT	S, O, C1, C2, C8	R4
7	SS7	*S. sonnei*	+	D	Vegetable salad	AZM, SPT, AMX, AMP, SXT	S, C1	R5
8	SS8	*S. sonnei*	+	D	Vegetable salad	TET, CST, NIT	S, O	R2
9	SS9	*S. sonnei*	+	D	Vegetable salad	SPT, AMC, AMX, SXT	S	R2
10	SS10	*S. sonnei*	+	D	Vegetable salad	NAL, SPT, AMC, KAN, AMX, NOR, AMP, TET, LVX, CHL, SXT	S, O, C1, C2, C8	R4
11	SS11	*S. sonnei*	+	D	Vegetable salad	AZM, SPT, AMX, AMP, SXT	S, C1	R5
12	SF12	*S. flexneri*	+	B	Vegetable salad	TET, CST, NIT	S, O	R2
13	SF13	*S. flexneri*	+	B	Vegetable salad	SPT, AMC, AMX, SPT, SXT	S	R2
14	SS14	*S. sonnei*	+	D	Ground meat	AZM, SPT, AMC, AMX, AMP, TET, CHL, SXT	S, C8, C9	R1
15	SS15	*S. sonnei*	+	D	Ground meat	AZM, IPM, NOR, TET, CST, SXT	S	R3
16	SS16	*S. sonnei*	+	D	Ground meat	NAL, SPT, AMC, AMX, NOR, AMP, TET, LVX, CHL, SXT	S, O, C1, C2, C8	R4
17	SS17	*S. sonnei*	+	D	Ground meat	AZM, SPT, AMX, AMP, SXT	S, C1	R5
18	SF18	*S. flexneri*	+	B	Ground meat	TET, CST, NIT, SXT	S, O	R2

Abbreviations: AMC, amoxicillin–clavulanic acid; AMP, ampicillin; AMX, amoxicillin; AZM, azithromycin; CHL, chloramphenicol; CST, colistin; IPM, imipenem; KAN, kanamycin; LVX, levofloxacin; NAL, nalidixic acid; NIT, nitrofurantoin; NOR, norfloxacin; SPT, streptomycin; SXT, trimethoprim–sulfamethoxazole; TET, tetracycline.

S, *bla*_SHV_; O, *bla*_OXA_; T, *bla*_TEM_; C1, *bla*_CTX‐M‐1_; C2, *bla*_CTX‐M‐1_; C8, *bla*_CTX‐M‐8_; C9, *bla*_CTX‐M‐9_

## 4 DISCUSSION

*Shigella* spp. is one of the most prominent foodborne bacterial pathogens worldwide, especially in developing countries such as India, Tunisia, Iran and Egypt, and 99% of shigellosis infections occur in these countries (Kotloff et al., 2018). Shigellosis is estimated to cause more than 80 million cases of hospitalizations and 700 000 deaths worldwide annually (Bennish & Ahmed, 2020). Significant numbers of shigellosis outbreaks are caused by consuming foods and water contaminated with different species of *Shigella* each year (Nygren et al., 2013). Since the human gastrointestinal tract is the main reservoir of *Shigella* species, vegetable salads (containing carrot, lettuce, and parsley), meat, and rarely dairy products which are usually being directly or indirectly contaminated with human feces have been introduced as the main sources of shigellosis outbreaks and foodborne diseases in different areas of the world. Several studies also reported contamination of these food items with *Shigella* spp. (Baker & The, 2018; Berthold‐Pluta et al., 2017; Nygren et al., 2013; Shahin et al., 2019). *Shigella* contamination of food products usually occurs by transmitting this pathogen from an infected person (usually food workers) using inappropriate processing techniques during food preparation (Puzari et al., 2018). Minimal information and studies are available about the current status of *Shigella* spp. in food products because this pathogen has not been routinely identified and isolated from food samples (Shahin et al., 2019).

In the current study, we isolated and estimated the prevalence rate of *Shigella* spp. in food products, including vegetable salad, raw cow milk, and ground meat samples collected in Qazvin, Iran, by using conventional culturing and serologic methods and determining antimicrobial susceptibility pattern and genetic diversity among the isolates using disk diffusion and RAPD‐PCR assays, respectively. From 405 food samples, 18 *Shigella* spp. were isolated and molecularly identified, indicating that a total of 4.4% of the samples were contaminated with different species of *Shigella*. The prevalence rate of *Shigella* spp. in this study is significantly more than the previous reports from Egypt (1.7%; 27 out of 1,600 samples)(Ahmed & Shimamoto, 2015), Tunisia (2.14%; 6 out of 280 samples)(Mokhtari et al., 2012), and Iran (1.4%; 19 out of 1,400 samples)(Shahin et al., 2019); and less than a report from Ethiopia (7.4%; 10 out of 135 samples)(Garedew et al., 2016). However, a study in Turkey reported that *Shigella* spp. were not detected in any food samples (Cetinkaya et al., 2008). These differences in prevalence rates between the studies may be due to the differences in geographical locations, sample size, source of sampling, and the level of public hygiene and health services. In addition to person‐to‐person transmission, foods are one of the primary vehicles for human infection with different species of *Shigella*. Consequently, travel‐ and trade‐associated *Shigella* may also cause a higher prevalence rate of this foodborne pathogen in different countries. Several epidemiological studies in these countries revealed various outbreaks caused by foodborne shigellosis were associated with consuming fresh food products, which are served raw, processed by hand, and exposed to a poor heat treatment (Ahmed & Shimamoto, 2015). Notably, the spread of *Shigella* contamination in foods may occur via flies, fingers, cutting surfaces, and utensils (Mokhtari et al., 2012).

In our study, the prevalence rate of *Shigella* spp. was higher in vegetable salads (3.20%) than in ground meat (1.23%) and raw cow's milk samples (0%). In an Egyptian study, Ahmed and Shimamoto (2014) reported a higher incidence of *Shigella* spp. in meat samples (2.0%) than in dairy products (1.4%)(Ahmed & Shimamoto, 2014). However, recently in Iran, Shahin et al. (2019) reported a higher incidence of *Shigella* spp. in vegetables (2.2%) than in ready‐to‐eat (2.0%) and meat samples (0.8%)(Shahin et al., 2019). Mokhtari et al. (2012) also demonstrated a higher prevalence rate of *Shigella* spp. in raw salad samples than in other food products in Tunisia (Mokhtari et al., 2012). Our results showed that *Shigella* spp. is not often recovered from dairy products comparable with the report from Turkey. Few publications reported *Shigella* spp. in dairy products or outbreaks of shigellosis in humans via consumption of these products (Shahin et al., 2019). Because humans and apes are regarded as the foremost and primary sources of *Shigella* spp., *Shigella* can be transferred to raw vegetables, vegetable salads, meat and dairy products by hand of food handlers, food processing workers, or food processing workers contaminated equipment (Ahmed & Shimamoto, 2015). A higher incidence of *Shigella* spp. in raw vegetable salads indicates the high potential of these products as *Shigella* carriers because of the utilization of sewage and wastewater for irrigation and human waste‐based fertilizers during the cultivation of edible vegetables (Ahmed & Shimamoto, 2014).

In our study, the frequency of *S. sonnei* (3.7%; 15 isolates) was higher than *S. flexneri* (0.74%; 3 isolates); however, *S. dysenteriae* and *S. boydii* were not detected in any sample. *S. flexneri* is the predominant species of *Shigella* caused shigellosis in developing countries. However, recent clinical and food contamination reports showed that *S. sonnei* had become the most prevalent species of *Shigella* in Iran (Karimi‐Yazdi et al., 2020). This revealed a correlation between the prevalence rate of frequent species of *Shigella* in food products and humans in Iran (Shahin et al., 2019). This correlation also was previously seen between clinical and food isolates of *Shigella* in Egypt (Ahmed & Shimamoto, 2015). It is worth noting that industrialization and improving the public hygiene level in developing countries such as Iran and Egypt may contribute to *S. sonnei* becoming the most prevalent species of *Shigella* in both food and clinical samples (Ahmed & Shimamoto, 2015; Shahin et al., 2019).

One of the most critical challenges in food safety is the emergence of antimicrobial‐resistant bacterial strains. Resistance genes could be transferred horizontally from animal food resources and environments to humans' normal flora and pathogens through food and drink chains (Caniça et al., 2019). A broad range of antibiotics is currently used in food animals for disease prevention, veterinary treatment, and growth promotion, allowing MDR foodborne bacterial pathogens (Ebmeyer et al., 2021). A previous study in Egypt showed a high level of resistance among *Shigella* spp. isolated from food samples against nalidixic acid, tetracycline, sulfamethoxazole/trimethoprim, ampicillin, chloramphenicol, cefotaxime, ceftriaxone, and ciprofloxacin (Ahmed & Shimamoto, 2015). A study which was conducted in Iran reported complete resistance to chloramphenicol, ampicillin, tetracycline, streptomycin, and trimethoprim and intermediate resistance to cefotaxime among the *Shigella* spp. isolated from clinical samples (Jomezadeh et al., 2014). Moreover, Jafari et al. (2009) isolated *Shigella* spp. from clinical samples in Iran with the highest resistance to sulfamethoxazole/trimethoprim, tetracycline, ampicillin, and chloramphenicol and susceptibility to cefotaxime and ceftazidime (Jafari et al., 2009). Another recent study by Shahin et al. (2019) in Iran indicated that *Shigella* spp. isolated from food samples were resistant to streptomycin (100%), tetracycline (98.5%), amoxicillin (78.9%), cephalothin (68.4%), cefuroxime (52.6%), and nalidixic acid (52.6%) (Shahin et al., 2019). A recent study by Bantawa et al. (2019) in Nepal showed high resistance to amoxicillin (100%), chloramphenicol (80%), tetracycline (60%), and nalidixic acid (20%) (Bantawa et al., 2019).

In our study, out of the *Shigella* isolates recovered, 83.3%, 66.6%, 66.6%, 61.1%, 50%, 50%, 50%, and 50% were resistant to sulfamethoxazole/trimethoprim, amoxicillin, streptomycin, tetracycline, ampicillin, amoxicillin–clavulanic acid, azithromycin, and chloramphenicol, respectively. One hundred percent were completely susceptible to cefoxitin, cefepime, amikacin, and gentamicin. These data indicate that there has been a considerable increase in resistance to different classes of antibiotics among the *Shigella* isolates. From a regional point of view, the results of the antimicrobial resistance patterns of the *Shigella* isolates from food products in this study are in accordance with the previous studies on food and clinical samples in Iran (Karimi‐Yazdi et al., 2020; Shahin et al., 2019). However, the communities and industries' reckless and unsupervised use of antibiotic agents to improve animal health may lead to differences between the antibiotic resistances patterns observed in studies in different countries (Ahmed & Shimamoto, 2014, 2015). The current study also indicated that all *Shigella* isolates showed an MDR phenotype. However, in previous studies on clinical isolates in Iran, the incidence rate of MDR *Shigella* spp. ranged from 45% to 100% (Karimi‐Yazdi et al., 2020). The prevalence rate of MDR *Shigella* spp. isolated from food samples was reported 90% in Egypt (Ahmed & Shimamoto, 2015). It is worth noting that the progressive increase in multidrug resistance among *Shigella* strains of food and clinical origins is considered a serious global concern and a threat to public health (Shahin et al., 2019). In this study, we detected beta‐lactamase genes including *bla*_SHV_, *bla*_OXA_, *bla*_CTX‐M‐1_, *bla*_CTX‐M‐1_, *bla*_CTX‐M‐8_, and *bla*_CTX‐M‐9_ genes in *Shigella* isolates. However, *bla*_TEM_ was not found in any isolate. Shahin et al. (2019) detected *bla*_TEM_, *bla*_SHV_, *bla*_CTX‐M‐15_, and *bla*_CMY_ antibiotic resistance genes among the *Shigella* isolates from different water samples (Shahin et al., 2019). Ahmed and Shimamoto (2015) detected *bla*_TEM_, *bla*_SHV_, *bla*_OXA_, *bla*_CTX‐M‐15_, *bla*_CTX‐M‐14_, *bla*_CTX‐M‐3_, and *bla*_CMY_ in *Shigella* spp. isolated from meat and dairy products. They found higher beta‐lactamase coding genes in meat than in dairy products (Ahmed & Shimamoto, 2015); however, we observed no significant difference between antibiotic resistance genes in the isolates from vegetable salads and ground meat samples. Beta‐lactamases are considered as the main enzymes responsible for resistance of gram‐negative bacteria such as *E. coli*, *Shigella*, etc., against the beta‐lactam antibiotics including cefoxitin, imipenem, amoxicillin, ampicillin, cefepime, and amoxicillin–clavulanic acid. The *bla*_SHV_ gene, which was the dominant beta‐lactamase gene in the *Shigella* isolates, encodes resistance against amoxicillin–clavulanic and amoxicillin antibiotics. Also, *bla*_OXA_ gene encodes resistance against ampicillin in *Shigella* isolates (Ahmed & Shimamoto, 2015).

The fluoroquinolones class of antibiotics, especially levofloxacin, norfloxacin, nalidixic acid, and ciprofloxacin, has been strongly considered the main treatment for shigellosis to the WHO guidelines for children and adults (Jafari et al., 2009). In the current study, 22.2%, 16.6%, and 16.6% of the MDR *Shigella* isolates were resistant to norfloxacin, nalidixic acid, and levofloxacin, respectively. Our results were lower than a similar study by Ahmed and Shimamoto (2015), which reported 95.8% of the MDR *Shigella* isolates resistant to nalidixic acid (Ahmed & Shimamoto, 2015). However, due to the development of antibiotic resistance, fluoroquinolone antibiotics are no longer effective for shigellosis (Karimi‐Yazdi et al., 2020). Colistin resistance phenotype has been observed in *Shigella* isolates, mostly in *S. flexneri* strains (Liang et al., 2018). In this study, we detected three isolates resistant to colistin, 2 of them were *S. flexneri,* and one was *S. sonnei*. The transfer of plasmid‐mediated colistin resistance into clinical bacterial strains can lead to colistin resistance in clinical *Shigella* isolates and other *Enterobacteriaceae* pathogens. It may be of concern that resistance against colistin was transferred, and other mobile genetic elements and other resistance and virulence factor encoding genes present in other parts of the plasmid or different plasmids may be transferred (Liang et al., 2018). However, this problem and its mechanism need more investigation in the future.

Several DNA fingerprinting methods have been developed and used to assess the genetic diversity and clonal relatedness of foodborne bacterial pathogens such as RAPD, ERIC, and BOX‐PCR methods (Kumar et al., 2018). We have used the RAPD‐PCR method to characterize the genetic diversity of *Shigella* isolates from vegetable salad and ground meat samples and assess the clonality of these isolates as other studies have previously demonstrated for clinical bacterial strains and foodborne pathogens (Staji et al., 2018). The result of RAPD‐PCR analysis showed a significant cluster (cluster R2) with six members constituting 33.3% of total *Shigella* isolates. All *S. flexneri* isolates recovered in this study were included in this cluster. All of the isolates in cluster R4 were strongly resistant to the standard antibiotics. All *Shigella* isolates included in cluster 4 were resistant to fluoroquinolones class of antibiotics, including levofloxacin, norfloxacin, and nalidixic acid. RAPD profiles demonstrated that the acquisition of resistance traits to fluoroquinolones might closely be associated with the acquisition of specific plasmids (Puzari et al., 2018). Wei et al. (2007) reported a close association between the antimicrobial resistance and PFGE genotyping profiles in *Shigella* strains of clinical origins (Wei et al., 2007). Zhu et al. (2017) also showed a significant association between antimicrobial resistance phenotypes and genotyping of *Shigella* spp. isolates from clinical samples (Zhu et al., 2017). So far, no studies have been performed on the relationship of antimicrobial resistance pattern and genotyping of *Shigella* spp. isolates from food products.

## 5 CONCLUSIONS

In this study, we demonstrated that the prevalence rate of *Shigella* spp. including *S. sonnei* and *S. flexneri* is more frequent in vegetable salad than other food samples. The prevalence rate of *S. sonnei* was higher than that of *S. flexneri*. However, *S. dysenteriae* and *S. boydii* were not recovered from the food samples. Also, no *Shigella* were detected in raw cow's milk samples. *Shigella* isolates were highly resistant to sulfamethoxazole/trimethoprim, amoxicillin, streptomycin, tetracycline, ampicillin, amoxicillin–clavulanic acid, azithromycin, and chloramphenicol; and highly susceptible to cefoxitin, cefepime, amikacin, and gentamicin antibiotics. All *Shigella* isolates were MDR to three or more classes of antibiotics. We detected *bla*_SHV_ resistance gene in all *Shigella* isolates; however, *bla*_TEM_ gene was not found in any isolate. High genetic diversity was observed among the *Shigella* isolates. A significant association was seen between antimicrobial resistance phenotypes and genotyping of *Shigella* isolates. The novel food control and monitoring systems, including surveillance of foodborne pathogens, especially MDR bacterial strains in developing countries, are required to detect, control, and prevent foodborne diseases such as shigellosis.

## CONFLICT OF INTEREST

All authors declare that they have no conflict of interest.

## Data Availability

We confirm that all data supporting the findings of this study are available within the article.
